# Systematic Review on Healthcare and Societal Costs of Tinnitus

**DOI:** 10.3390/ijerph18136881

**Published:** 2021-06-26

**Authors:** Ilias Trochidis, Alessandra Lugo, Elisa Borroni, Christopher R. Cederroth, Rilana Cima, Dimitris Kikidis, Berthold Langguth, Winfried Schlee, Silvano Gallus

**Affiliations:** 1 ViLabs, Limassol 3030, Cyprus; it@vilabs.eu; 2 Department of Environmental Health Sciences, Istituto di Ricerche Farmacologiche Mario Negri IRCCS, 20156 Milan, Italy; alessandra.lugo@marionegri.it (A.L.); borroni_elisa@libero.it (E.B.); silvano.gallus@marionegri.it (S.G.); 3 Laboratory of Experimental Audiology, Department of Physiology and Pharmacology, Karolinska Institutet, 171 77 Stockholm, Sweden; christopher.cederroth@ki.se; 4 National Institute for Health Research (NIHR) Nottingham Biomedical Research Centre, Nottingham University Hospitals NHS Trust, Ropewalk House, Nottingham NG1 5DU, UK; 5 Division of Clinical Neuroscience, Hearing Sciences, School of Medicine, University of Nottingham, Nottingham NG7 2UH, UK; 6 Faculty of Psychology and Educational Science, Health Phycology, KU Leuven University, B-3000 Leuven, Belgium; rilana.cima@kuleuven.be; 7 Centre of Knowledge and Expertise in Rehabilitation and Audiology, Tinnitus Center of Expertise, Adelante, 6432 CC Hoensbroek, The Netherlands; 8 Faculty of Psychology and Neurosciences, Experimental Health Psychology, Maastricht University, P.O. Box 616, 6200 MD Maastricht, The Netherlands; 9 First Department of Otorhinolaryngology Head and Neck Surgery, National and Kapodistrian University of Athens, Hippocrateion General Hospital, 11527 Athens, Greece; dimitriskikidis@yahoo.com; 10 Department of Psychiatry and Psychotherapy, University of Regensburg, 93053 Regensburg, Germany; berthold.langguth@medbo.de

**Keywords:** tinnitus, healthcare cost, economic burden, cost analysis, systematic review

## Abstract

Tinnitus disability is a heterogeneous and complex condition, affecting more than 10% and compromising the quality of life of 2% of the population, with multiple contributors, often unknown, and enigmatic pathophysiology. The available treatment options are unsatisfactory, as they can, at best, reduce tinnitus severity, but not eliminate its perception. Given the spread of tinnitus and the lack of a standardized treatment, it is crucial to understand the economic burden of this condition. We conducted a systematic review of the literature on PubMed/MEDLINE, Embase, the Cochrane Database of Systematic Reviews (CDSR) and Google Scholar, in order to identify all the articles published on the economic burden of tinnitus before 1 April 2021 (PROSPERO—International prospective register of systematic reviews—No: CRD42020180438). Out of 273 articles identified through our search strategy, only five articles from studies conducted in the United States of America (USA), the Netherlands and the United Kingdom (UK) provided data on tinnitus’s economic costs. Three studies provided mean annual estimates per patient ranging between EUR 1544 and EUR 3429 for healthcare costs, between EUR 69 and EUR 115 for patient and family costs and between EUR 2565 and EUR 3702 for indirect costs, including productivity loss. The other two studies reported an annual mean cost of EUR 564 per patient for tinnitus-related clinical visits, and total costs of EUR 1388 and EUR 3725 for patients treated with a sound generator and Neuromonics Tinnitus Treatment, respectively. Our comprehensive review shows a gap in the knowledge about the economic burden of tinnitus on healthcare systems, patients and society. The few available studies show considerable expenses due to healthcare and indirect costs, while out-of-pocket costs appear to be less financially burdensome. Comprehensive health economic evaluations are needed to fill the gaps in current knowledge, using a unified method with reliable and standardized tools.

## 1. Introduction

Tinnitus is a continuous auditory perception in the ears or head in the absence of a corresponding external stimulus [1]. Tinnitus can become a chronic health problem, and it affects 10–15% of the general adult population, with 1–5% of adults severely impacted [1,2,3,4,5]. Tinnitus prevalence increases with older age, reaching up to 40% among the elderly [2,3]. The incidence of tinnitus is expected to grow as a consequence of the increase in industrialization worldwide and a longer lifespan [6]. Approximately 10% of subjects with tinnitus develop mild to severe disability. Severe disabling tinnitus, being strongly associated with depression, anxiety, insomnia, difficulty concentrating and poor psychological well-being, significantly negatively impacts quality of life [2,3,4,5,6].

When disabling and impacting daily functioning, tinnitus is a heterogeneous and complex condition with multiple associated biological, psychological and contextual contributors, which often remain unknown [7]. Moreover, with increasing severity, it appears to segregate in families, suggesting a genetic cause as well [8,9]. The available treatment options are unsatisfactory [10,11]. Multiple treatments have been proposed for tinnitus, and many of them have shown efficacy in a subgroup of patients [12]. Still, it is currently difficult to predict the optimal and most promising treatment for an individual tinnitus patient. The few evidence-based treatment options efficacious for every tinnitus are aimed at ameliorating the disability caused by tinnitus rather than eliminating the perception [13]. Indeed, the most widespread of these management therapies consist of auditory stimulation and cognitive-behavioral therapy, aiming at modulating mechanisms that would otherwise maintain tinnitus disability [5,6].

Consequently, people suffering from this condition try out various possible treatments, one after the other [12], and refer to a variety of caregivers in unstructured and non-standardized ways, in the hope of finding proper treatment [14]. This creates high costs both for patients and healthcare systems and complicates cost evaluations, facing several challenges when aiming to include all the treatment pathways followed by patients. Indeed, over 4 million prescriptions are written every year in Europe and the USA for tinnitus relief, but these are all off-label prescriptions from a wide variety of therapeutic drugs with uncertain efficacy [5,6], even though there is a recommendation against medication for tinnitus in the European guidelines [15].

Thus, tinnitus management carries a significant financial burden for both healthcare systems and society, including patients [16]. Expenses related to the management of tinnitus are not limited to direct medical costs (e.g., specialist’s visits, purchasing drugs or other medical devices) but also include individual indirect costs such as travel expenses, costs for recommended activities (such as sport or meditation), costs for family members and loss of income because of reduced working capabilities. Tinnitus frequently leads to a work-related disability, often resulting in compensation of payments. For example, in 2012, the United States Department of Veterans Affairs (VA) spent USD 1.2 billion on tinnitus-related compensation to veterans [16]. Moreover, in many cases, tinnitus causes sick leave and disability pension [17,18]. Thus, the financial burden tinnitus imposes on governments and industries is significant [6]. Moreover, tinnitus correlates with impaired quality of life, which could be further estimated in terms of costs. Indeed, at least two studies showed that high healthcare costs were substantial in terms of quality-adjusted life years (QALYs) per person [19,20].

Given the spread of tinnitus and the currently still relatively low investment in tinnitus research [21], which leads to a lack of standardized treatment for this condition, it is crucial to understand the exact economic burden of tinnitus. This study aims to conduct a systematic review to collect information on both direct (medical and non-medical costs) and indirect costs (including societal costs, such as work loss and reduced productivity) for tinnitus management from the available scientific literature. This will allow us to describe the costs for the healthcare system and tinnitus patients under the current practice. The results of this systematic review will help us understand and evaluate the extent of evidence currently available on the financial burden of tinnitus and identify potential gaps to direct future research.

## 2. Materials and Methods

### 2.1. Data Sources and Search Strategy

The following scientific databases were considered to conduct this systematic literature search: PubMed/MEDLINE, Embase and the Cochrane Database of Systematic Reviews (CDSR). A search on Google Scholar was also performed in order to identify relevant articles published in scientific journals not indexed in those databases. In addition, the databases of the World Health Organization (WHO), Eurostat and the Organisation for Economic Co-operation and Development (OECD) were consulted to retrieve possible additional relevant information about direct and indirect costs associated with tinnitus and related quality-adjusted life years. The search strategy was designed for PubMed and then adapted for use in the other databases. Search terms included terms for the condition and the considered outcome (i.e., “tinnitus AND (economic OR costs OR market OR “Cost of Illness” OR QALY)). We did not apply any restriction on publication time, considering all scientific articles published in English before the search date. Articles published in a language other than English were excluded from the review. Reference lists of other reviews were also checked to identify other potentially relevant publications.

On 1 April 2021, we applied the search strategy. Overall, through PubMed, 137 publications were identified, with an additional 14 on Embase, 28 on CDSR and 170 publications on Google Scholar. Excluding duplicates, we obtained a total of 273 publications (Supplementary Figure S1).

### 2.2. Eligibility Criteria

In order to be eligible, studies had to provide data on direct or indirect costs due to the management of tinnitus disability in human subjects. We excluded studies on animals or biological tissues. We did not apply restrictions on the type of study design; thus, studies considered for inclusion were: cross-sectional studies (including population-based surveys on specific sub-populations), case–control studies, cohort studies, clinical trials and case series. Both original articles and reviews were considered, while unpublished studies, conference abstracts and proceedings, dissertations, theses and, more generally, non-peer-reviewed papers were not considered.

### 2.3. Study Selection

We combined those publications detected on various databases in an EndNote library (i.e., a software for reference management). In the first screening, two reviewers evaluated titles and abstracts independently to identify publications that met our inclusion criteria. The reviewers independently assigned scores 1–5 to the articles as follows: 1 = Publication not pertinent or of limited interest for our review; 2 = Publication probably not pertinent or of limited interest; 3 = Not possible to evaluate based on title/abstract/keywords only; 4 = Publication probably pertinent or of interest; 5 = Publication pertinent or of clear interest for our review. After the first screening, the two reviewers’ scores were added, obtaining total scores ranging from 2 to 10. A publication with a combined score of 4 or less was not considered for further evaluation. In the second screening, the full text of all the potentially eligible studies was retrieved and independently assessed for eligibility by two reviewers.

### 2.4. Data Extraction

For each publication satisfying the eligibility criteria, we collected: (a) general information on the publication (first author, year of publication, journal), (b) study characteristics (country, calendar period, study design, sample size), (c) data on direct and indirect costs for tinnitus management, and (d) the model used to compute the estimates (including adjustments). In order to allow for comparisons, all costs were converted from the local currency into EUR (conversion rate: USD 1 = EUR 0.85; GBP 1 = EUR 1.10). Data extraction was performed using Microsoft Excel and EndNote X7.

## 3. Results

Out of 273 identified publications, 26 were included after the first screening of titles and abstracts. Of these, only five articles met the eligibility criteria and were included in the present systematic review. No relevant additional information was found in the databases of the WHO, Eurostat and OECD. Table 1 shows the description of eligible included studies. Two studies were conducted in the USA: a cross-sectional study from 2011, analyzing a sample of 692 tinnitus patients, estimated that the annual average tinnitus-related costs per patient for clinical visits were EUR 564 (standard deviation, SD: EUR 1186) [22]; a cohort study evaluated the treatment costs during a 1-year period of follow-up of 56 tinnitus patients, treated either with sound generators or the Neuromonics Tinnitus Treatment. Treatment costs were EUR 1388 for sound generators and EUR 3725 for the Neuromonics Tinnitus Treatment [23].

A cross-sectional study from the Netherlands provided direct and indirect annual mean costs per patient. Total annual mean healthcare costs added up to EUR 1544, patient and family costs to EUR 69 and costs due to productivity loss to EUR 3702 [7]. This study reported higher costs for patients with severe compared to moderate and mild tinnitus. In particular, healthcare costs were EUR 767, EUR 1329 and EUR 2218 for patients with mild, moderate and severe tinnitus, respectively. The corresponding estimates for patients and family costs were EUR 31, EUR 61 and EUR 115, and those due to productivity loss were EUR 1222, EUR 4781 and EUR 5105.

A randomized controlled trial (RCT) from the Netherlands conducted in 2007–2011 on 492 tinnitus patients estimated annual mean direct and indirect costs per patient divided by usual care (UC) and specialized care (SC). The study provided healthcare costs (UC: EUR 3300; SC: EUR 3429), patient and family costs (UC: EUR 115; SC: EUR 90) and costs due to a loss of productivity (UC: EUR 2565; SC: EUR 2764). Thus, estimates for the total annual costs (i.e., the sum of the three categories mentioned above) were EUR 5980 for UC and EUR 6283 for SC [19].

The last eligible included study was a cohort study conducted in the UK, estimating mean annual direct costs expressed as healthcare costs only. The mean annual total healthcare costs were EUR 1938 [20]. Indirect costs and patient and family costs were not provided in this publication.

**Figure 1:**
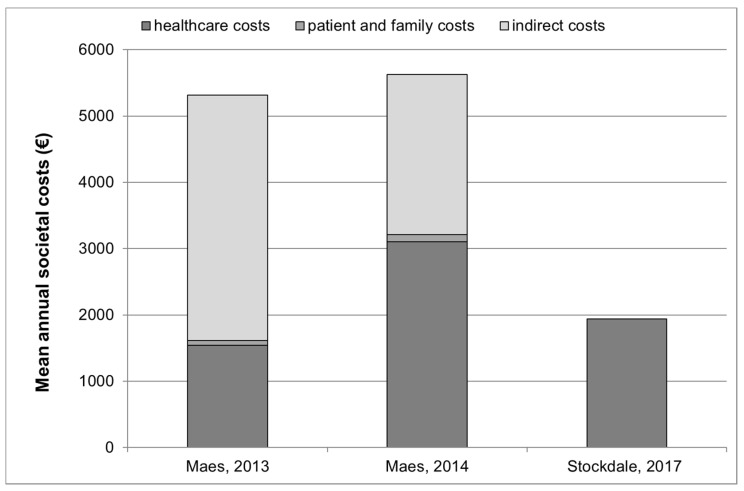
Annual healthcare, patient and family and indirect costs (where available *) for tinnitus management in 3 studies °. * The study by Stockdale et al., 2017 [20] provided information on healthcare costs only. Indirect costs and patient and family costs were not provided in this publication. ° Studies by Goldstein et al., 2015 [22] and Newman and Sandrige, 2012 [23] are not presented in this figure since they did not provide an estimate of overall societal or healthcare costs but only provided estimates for clinical visits, and for sound generators and Neuromonics Tinnitus Treatment.

The costs identified in studies that provided healthcare, patient and family and indirect costs are graphically presented in Figure 1.

## 4. Discussion

### 4.1. Main Findings

This is the first systematic review summarizing direct and indirect costs for tinnitus management in the current practice, based on the published scientific literature. Although there is a wide consensus among scientific and clinical professionals that tinnitus is associated with high direct and indirect healthcare costs, our systematic review identified only five studies that calculated costs for tinnitus management [7,19,20,22,23], with only three of them reporting overall estimates of societal or healthcare costs [7,19,20]. Among the latter three studies, the mean annual estimates per patient ranged between EUR 1544 and EUR 3429 for healthcare costs, between EUR 69 and EUR 115 for patient and family costs (i.e., out-of-pocket costs) and between EUR 2565 and EUR 3702 for indirect costs (i.e., costs due to the loss of productivity). The other two studies reported an annual mean cost of EUR 564 per patient for tinnitus-related clinical visits [22], and total costs of EUR 1388 and EUR 3725 for patients treated with sound generators and the Neuromonics Tinnitus Treatment, respectively [23]. These costs have been considered comparable to those observed for unexplained pain [20].

### 4.2. Cost Categories

One aspect that emerged from most studies in the scientific literature and other relevant databases was the lack of comprehensive and detailed information on tinnitus costs, except for two studies from the Netherlands. Cost categories ideally include both direct costs (direct medical and non-medical costs) and indirect costs, including societal costs (e.g., work loss, worker replacement, reduced productivity from illness and disease, family costs and financial estimation of the impact on quality of life) [20,24]. This systematic review identified only two studies, both from the Netherlands, providing a detailed description of direct costs (healthcare, patient and family costs) and indirect costs (due to productivity losses) [7,19]. Two additional studies provided only a portion of the overall picture of costs, showing total healthcare costs [20] and costs for clinical visits [22].

### 4.3. Country Differences

In our systematic review, two of the studies reporting information on the economic burden of tinnitus were conducted in the US [22,23], two in the Netherlands [7,19] and one in the UK [20]. Therefore, it is evident that data on healthcare and societal costs of tinnitus come from a very limited portion of countries worldwide. The current scientific literature does not permit comparison among countries, since complete information on total annual average costs for tinnitus patients comes from the Netherlands only [7,19]. To our knowledge, no cross-border study comparing cost data in different countries, applying the same methodology for calculating tinnitus costs, is available. This type of study would allow investigating the impact of different healthcare systems, therapy procedures, treatment availability and countries’ economic indicators on tinnitus-related costs.

However, an attempt for a broad estimation of the total costs in the EU could pursue the following path: The first step would be to broadly translate expenses to the average income of these countries and then consider the average EU income. The second step would be to extend these estimates to the whole EU, taking into account that various epidemiological studies estimated a prevalence of tinnitus exceeding 10%, which means that at least 30 million people in the EU live with tinnitus.

Additionally, comparisons between countries on the costs of various treatments for tinnitus management are lacking in the current literature. For future health economic evaluations of treatments for disabling tinnitus, consensus on a set of standardized and homogenous evaluative tools is of high importance, in order to facilitate cross-country and cross-study comparisons.

### 4.4. Impact of Patients’ and Tinnitus Characteristics (e.g., Severity) on Costs

Only one study conducted in the Netherlands investigated the impact of tinnitus severity on costs, showing that the more disabled patients had significantly higher healthcare costs compared to patients with mild to moderate complaints [7]. In fact, patients with severe tinnitus disability had more contacts with the GP, medical specialists—including ENT specialists and neurologists—and other healthcare professionals such as psychologists, social workers and clinical physicists in audiology [7]. This study also found that productivity losses were higher for moderate and severe tinnitus patients, compared to the mild group. At the same time, no differences regarding out-of-pocket costs were observed across the three groups [7]. The same study identified other relevant predictors for both higher healthcare and societal costs, besides tinnitus severity. These included younger age, shorter duration of tinnitus (less than 1 year) and higher scores of depression, while sex, level of education, health-related quality of life and anxiety did not impact costs [7]. No information on the effect of other possibly relevant socio-demographic, economic and clinical characteristics is available in the current scientific literature. Including these predictors in other populations [20] might provide insight into the effect of socio-demographic and economic parameters, such as family and personal socio-economic status, and tinnitus-related characteristics on costs.

### 4.5. Strengths and Limitations of the Review

To our knowledge, this is the first systematic review providing a synthesis of the healthcare and societal costs of tinnitus at an individual level in the scientific literature. Strengths of this review include the comprehensive search strategy conducted on a large set of scientific databases, including PubMed/MEDLINE, Embase and the CDSR. Moreover, an additional search on Google Scholar and databases of the WHO, Eurostat and OECD was conducted in order to check for relevant articles published in scientific journals not indexed in the previously mentioned databases. The detailed procedure used for selecting articles and data extraction further assures the qualitative value of this systematic review. Limitations include the relatively small number of included studies, leading to low generalizability of results across countries and across patient groups.

### 4.6. Future Perspectives and UNITI Project

This systematic review found only a few studies dealing with the economic burden of tinnitus, none of which were published in recent years (2018–2021). Thus, it is important to be able to make some updated and validated statements about the economic impact of tinnitus on the individual and society. Future studies are needed to provide new detailed data on the healthcare and societal costs for tinnitus. Such studies are useful today because of their implications for health policy (e.g., determining priorities by reporting tinnitus cases that require special help, evaluating the efficacy of treatments, allocating appropriate funds for research and development of tinnitus treatments). The framework for calculating tinnitus costs must include data from various countries (see Section 4.3) and must consider individual-level characteristics of tinnitus patients (e.g., sex, age, income, concomitant comorbidities) and of the symptom itself (e.g., tinnitus onset, duration, severity) that could determine higher costs for tinnitus management (see Section 4.4). At the same time, information on costs per quality-adjusted life years (QALY) is scant in the literature. The UNITI project provides the opportunity to add to the gaps in knowledge on the economic burden of tinnitus (https://uniti.tinnitusresearch.net accessed on 1 April 2021). Within UNITI, a large multicenter randomized controlled trial will be carried out in five clinical centers across Europe. The cost analysis methodology of the studies from the Netherlands [7,19] will be adopted and applied, and patient- and tinnitus-specific characteristics relevant to tinnitus costs will be collected on all patients entering the RCT. The UNITI consortium has committed to performing a comprehensive health economic evaluation, including an analysis of cross-country differences.

## 5. Conclusions

The current status of the scientific literature highlights a serious lack of studies estimating, in detail, the economic burden of tinnitus on healthcare systems, patients, their families and society. Available studies show considerable expenses due to healthcare costs and indirect costs—mainly costs for productivity loss—while out-of-pocket costs appear to be less financially burdensome. Findings also indicate a direct relationship between tinnitus severity and related costs. Insights from existing studies point to several under-investigated but tinnitus-relevant predictors, such as country of origin, socio-economic factors and individual patients’ sex, age, educational level and tinnitus severity and duration. Identifying the economic burden of tinnitus in various cost categories is crucial to better understand tinnitus healthcare organization and treatment implementation in current practice in various countries and consequently reduce unnecessary costly and ineffective treatment strategies for patients and healthcare systems.

## Figures and Tables

**Table 1:** Description of eligible studies included in this systematic review.

First Author, Year [reference]	Study Design	Country (Study Period)	Tinnitus Cost Determination	Study Population	Outcome Measures	Estimates
Goldstein, 2015 [22]	Cross-sectional	USA (2011)	Costs were estimated through local hospital data stores	Sample of 692 patients with subjective tinnitus who visited the clinic in 2011	Total annual clinical costs for tinnitus-related visits	Estimates are provided in annual mean per person (SD) Annual tinnitus-related cost for clinical visits only: USD 663 (USD 1395) (i.e., EUR 564 (EUR 1186))
Newman, 2012 [23]	Cohort	USA (not specified)	Cost estimates were based on patient charges for each treatment option	56 patients enrolled from a tinnitus clinic: 23 treated with SG, 33 with NTT	Healthcare costs for 1 year of two specific treatments	SGs total costs: USD 1633 (i.e., EUR 1388) NTT total costs: USD 4382 (i.e., EUR 3725)
Maes, 2013 [7]	Cross-sectional	Netherlands (not specified)	Costs were estimated using a self-administered questionnaire with a recall period of 3 months. The mean costs were then multiplied by 4 to obtain annual mean costs per patient.	Sample of 492 patients referring to an audiological secondary-care facility	Mean annual costs divided into three categories: healthcare costs, patient and family costs and indirect costs	**Healthcare costs:** EUR 1544 These costs include those for GP practice (EUR 225; visit, home visit, assistant visit, weekend and evening), medical specialists (EUR 771; ENT specialist visit, dental surgeon visit, neurologist visit, other medical specialists), other healthcare professionals (EUR 527; clinical physicist in audiology, physiotherapist, psychologist, psychiatrist, social worker, occupational therapist, company doctor, homeopath, acupuncturist, haptonomist, magnetizer), prescribed medication (EUR 21). **Patient and family costs:** EUR 69 These costs include over-the-counter medication, traveling expenses, sports, medication, ear candle, other costs. **Productivity loss** (loss of productivity at paid labor): EUR 3702 **Total annual costs:** EUR 5315
Maes, 2014 [19]	RCT	Netherlands (2007–2011)	Costs were estimated from the exact amount of care consumed at the audiologic center by each patient, from the GIP databank 2009 and from the information of the Dutch Association of Hearing Aid Dispensers.	492 tinnitus patients who referred to the audiologic center and were randomized to SC or UC	Mean annual costs divided into three categories: healthcare costs, patient and family costs and indirect costs	**Healthcare costs:** UC: USD 3882 (i.e., EUR 3300) and SC: USD 4034 (i.e., EUR 3429) These costs include first-level tinnitus care (UC: USD 1848 and SC: USD 2091; pure tone audiometry, speech audiometry, tympanometry, tinnitus analysis, uncomfortable loudness levels, individual consult by clinical physicist in audiology, hearing aid fitting, new hearing aid, hearing aid check and optimization, fitting tinnitus masker, new tinnitus masker, BERA, intake psychologist, tinnitus educational group session), second-level tinnitus care (UC: USD 365 and SC: USD 694; individual trajectory, treatment group A, treatment group B, social work trajectory), general practitioner practice (UC: USD 166 and SC: USD 97; GP visit, GP home visit, GP assistant visit, GP weekend and evening), hospital care (UC: USD 562 and SC: USD 479; ENT specialist visit, neurologist visit, dental surgeon visit, other medical specialist), other healthcare professionals (UC: USD 941 and SC: USD 674; physiotherapist, psychologist, psychiatrist, social worker, occupational therapist, company physician, homeopath, acupuncturist, haptonomist, magnetizer), prescribed medication. **Patient and family costs:** UC: USD 135 (i.e., EUR 115), SC: USD 106 (i.e., EUR 90) These are divided into: over-the-counter medication, traveling expenses, sports, medication or other costs. **Productivity loss** (loss of productivity at paid labor): UC: USD 3018 (i.e., EUR 2565), SC: USD 3252 (i.e., EUR 2764) **Total annual costs:** UC: USD 7035 (i.e., EUR 5980) SC: USD 7392 (i.e., EUR 6283)
Stockdale, 2017 [20]	Cohort	UK (not specified)	A cost model was constructed considering the most common treatment pathways and was applied to a cohort of patients	Cohort of patients referring to a GP for tinnitus for the first time	Annual healthcare treatment costs	Digital hearing aids: GBP 85 Hearing aids assessments: GBP 65 Hearing aid fitting: GBP 65 Hearing aid follow-up: GBP 108 Hearing aid batteries: GBP 12 Hearing aid repairs: GBP 52 CBT: GBP 471 Tinnitus therapy plus wearable sound generator: GBP 303 GP session: GBP 52 Pharmacotherapy—betahistine: GBP 25 Pharmacotherapy—amitriptyline: GBP 13 MRI: GBP 85 Associate medical specialist in ENT: GBP 121 Audiologist (1 h): GBP 19 Clinical psychologist (2 h): GBP 286 **Total healthcare costs:** GBP 1762 (i.e., EUR 1938)

BERA: brainstem evoked response audiometry; CBT: cognitive-based therapy; ENT: ear, nose and throat; GP: general practitioner; MRI: magnetic resonance imaging; NTT: Neuromonics Tinnitus Treatment; RCT: randomized controlled trial; SC: standard care; SD: standard deviation; SG: sound generator; UC: usual care.

## Data Availability

The data presented in this study are available in Section 3, in Table 1, in Figure 1 and in Figure S1.

## Supplementary Materials

The following are available online at https://www.mdpi.com/article/10.3390/ijerph18136881/s1, Figure S1: Flowchart of the systematic review.
