# Supplementary material for: Systematic Review on Healthcare and Societal Costs of Tinnitus
Source: Int J Environ Res Public Health. 2021 Jun 26;18(13):6881. doi: 10.3390/ijerph18136881 (PMC8297244; doi:10.3390/ijerph18136881)
Supplement: Supplementary file 1 [file ijerph-18-06881-s001.zip › ijerph-1229284-supplementary.pdf]

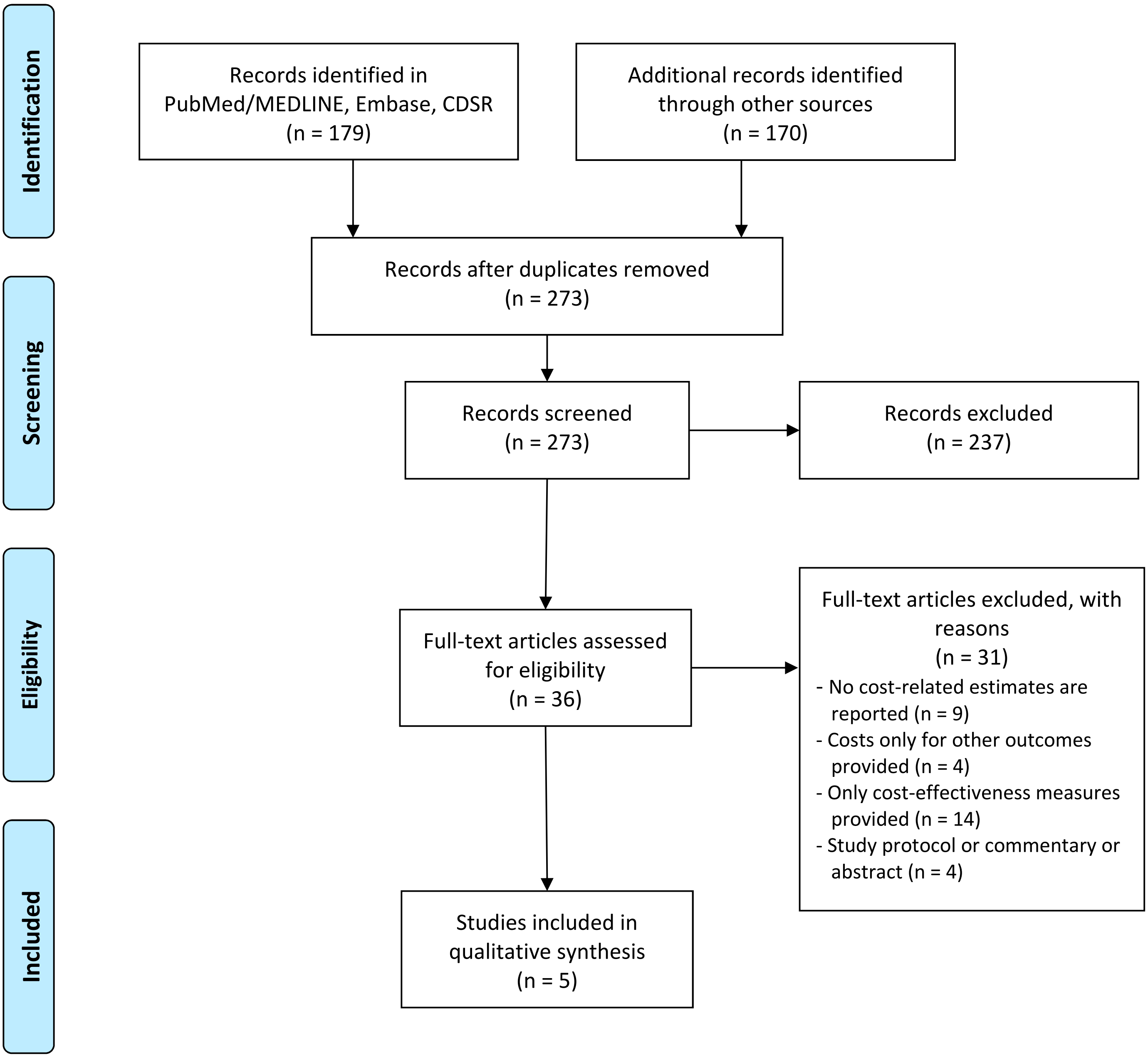

Figure S1 Flowchart of the systematic review.

From: Moher D, Liberati A, Tetzlaff J, Altman DG, The PRISMA Group (2009). *P*referred *R*eporting *I*tems for *S*ystematic Reviews and *M*eta-*A*nalyses: The PRISMA Statement. PLoS Med 6(7): e1000097. doi:10.1371/journal.pmed1000097

For more information, visit www.prisma-statement.org.
